# Increased Serum Level of Interleukin-10 Predicts Poor Survival and Early Recurrence in Patients With Peripheral T-Cell Lymphomas

**DOI:** 10.3389/fonc.2020.584261

**Published:** 2020-10-13

**Authors:** Yan Zhang, Yanlong Zheng, Lihong Shou, Yuanfei Shi, Huafei Shen, Mingyu Zhu, Xiujin Ye, Jie Jin, Wanzhuo Xie

**Affiliations:** ^1^Department of Hematology, The First Affiliated Hospital, College of Medicine, Zhejiang University, Hangzhou, China; ^2^Department of Hematology, Huzhou Central Hospital, Affiliated Cent Hospital of Huzhou University, Huzhou, China

**Keywords:** peripheral T-cell lymphoma, interleukin-10, complete response, prognosis, early recurrence

## Abstract

Peripheral T cell lymphoma (PTCL) is an alloplasm group of aggressive and lymphoproliferative tumors with heterogeneous morphological changes of mature T cell immunophenotype. It has multiple subtypes and most of them have poor prognosis. Interleukin 10 (IL-10) is one kind of multi-cell-derived and multifunctional cytokine. It regulates the growth and differentiation of cells, participates in inflammation and immune response, plays an important role in tumor and infection, and is closely related to blood system diseases. Therefore, we implemented a retrospective study of 205 patients who were newly diagnosed with PTCL to explore the relationship between IL-10 and prognosis and early recurrence. We found patients with IL-10 ≥3.6 pg/ml achieved a lower CR rate and higher 1-year recurrence rate than patients with IL-10 <3.6 pg/ml (14.4 vs. 51.9%; 17.6 vs. 49.5%). On multivariate analysis, moreover, elevated IL-10 is an extremely important prognostic factor in PTCL, which can lead to worsening of overall survival (OS), low complete response (CR) rate and higher early relapse rate. Therefore, measurement of IL-10 levels in peripheral blood at the initial stage are useful for predicting the prognosis and helping us to make different treatment plans for individual patients. In the near future, IL-10 inhibitors or antagonists may become a new method of immunotargeting therapy for patients with PTCL.

## Introduction

Peripheral T cell lymphoma (PTCL) is a group of rare lymphoid malignancies with heterogeneous morphological that originating from post-thymic T cells or mature natural killer (NK) cells, accounting for about 10% of all non-Hodgkin’s lymphomas (NHL) in Western countries (1). However, the morbidity of PTCL is higher in Asia, it makes up 25–30% of NHL in China (2). According to the WHO classification, PTCL can be further distinguished based on their immunophenotypical, morphological, biological, and clinical features (3). The most common subtypes include PTCL-not otherwise specified (PTCL-NOS), extra-nodal natural killer (NK)/T cell lymphoma, nasal type (ENKTL), angioimmunoblastic T-cell lymphoma (AITL), anaplastic large-cell lymphoma (ALCL), include ALCL anaplastic lymphoma kinase positive(ALCL, ALK +), and ALCL anaplastic lymphoma kinase negative (ALCL, ALK-). Relatively uncommon subtypes include monomorphic epitheliotropic intestinal T-cell lymphoma (MEITL), T-cell Large Granular Lymphocytic Leukemia (T-LGLL), subcutaneous panniculitis like T-cell lymphoma (SPTCL), mycosisfungoides/Sezary’s syndrome (MF/SS), Hepatosplenic T-cell lymphoma (HSTCL), and so on. The overall characteristics of them are aggressive clinical course and poor response to therapy. Due to its rarity and heterogeneity, prognostic studies on PTCL are relatively scarce.

The development of science and technology as a whole society has promoted the development of molecular biology and the understanding of PTCL is greatly improved, particularly in tumor micro-environment. Interleukin 10 (IL-10) is one kind of multi-cell-derived and multifunctional cytokine. It regulates the growth and differentiation of cells, participates in inflammation and immune response, plays an important role in tumor and infection, and is closely related to blood system diseases. It is mainly excreted by activated T cells, monocytes, B cells, macrophages, certain tumor cells and so on. Not only that, various studies have shown that IL-10 can promote the development of tumor cells. Studies have shown that some malignant T cells can grow and survive well, but they are mainly closely related to (M2) macrophages that are alternately activated within the microenvironment. Moreover, IL-10 will have an enhanced effect to some extent (4). In addition, the abnormality of JAK/STAT pathway will promote IL-10-mediated lymphoid hyperplasia by M2 macrophages (5). The GATA-binding protein 3 (GATA3) is a transcription factor related to the type 2 helper (Th2) cell. If GATA3 is overexpressed, it will affect the survival rate of PTCL, to be precise, it will reduce the survival rate (6). Not only that, it also induces the expression of Th2-related cytokines, such as IL-10 (7). In this way, IL-10 has more ways to help malignant lymphocytes express genetic material, achieve the purpose of reproduction, and promote immune escape of tumor. Because previous studies had shown that IL-10 is associated with abnormal proliferation of cancer cells in a variety of cancers (breast cancer, cervical cancer, thyroid cancer, etc.) (8–10). Moreover, the role of IL-10 in Hodgkin’s lymphoma, diffuse large B-cell lymphoma (DLBCL) and Burkitt’s lymphoma had also been extensively studied (11, 12). However, little is known about the effects of IL-10 on response and prognosis, especially the early recurrence or progression of PTCL patients. Consequently, we retrospectively analyzed 205 PTCL patients, and aimed to study the significance of IL-10 in their treatment response, survival rate, and early relapse.

The writing of our article refers to the Reporting Recommendations for Tumor Marker Prognostic Studies (REMARK): Explanation and Elaboration guidelines (13).

## Materials and Methods

### Patients and Control Subjects

This is a single-center retrospective cohort analysis of 205 newly diagnosed PTCL patients at the First Affiliated Hospital of Zhejiang University School of Medicine from January 2014 to June 2019. The inclusion criteria for this retrospective study were as follows: (1) Age ≥18 years; (2) Pathological diagnosis of PTCL; (3) No long-term glucocorticoid treatment or chemotherapy before collecting clinical data; (4) Complete Clinical data. According to the Helsinki statement, all patients in our study signed an informed consent form before peripheral blood was drawn. Although the treatment regimens of PTCL patients in our study were inconsistent, most patients received combination chemotherapy, including cyclophosphamide-doxorubicin-vincristine-prednisone (CHOP) or CHOP-like regimens, and all the ENKTL patients’ chemotherapy containing Pegaspargase. The final observation time was January 2020, and the median follow-up time was 14 months.

### Measurement and Evaluation Indicators

We reviewed medical records, physical examinations, laboratory results, pathology reports, and radiological findings to reanalyze the clinical data of these patients. The following experimental and clinical data were collected: age at diagnosis, sex, International Prognostic Index (oncology) (IPI) score, Eastern Cooperative Oncology Group (ECOG), Ann Arbor stage, B symptoms (fever, night sweating, or weight loss), serum albumin level, bone marrow involvement, Hemophagocytic syndrome (HPS), serum interleukin-6 level (IL-6), serum interleukin-10 level (IL-10), serum interferon γ level (IFN γ), Epstein-Barr virus(EBV) infection, number of involved extra-nodal sites, serum lactate dehydrogenase (LDH), beta-2 micro-globulin(β2-MG) and serum ferritin (SF). Regular imaging examinations were performed after treatment, and disease status were recorded and analyzed. Follow-up was performed by making phone calls or reviewing medical records. Progression was defined as an increase in volume of the original focus or the development of a new focus. Early recurrence was defined as relapse or progressive disease occurrence within 1-year post-chemotherapy. OS was defined as the time from diagnosis to death for any reasons or last follow-up.

### Detection of Cytokines in Peripheral Blood of Patients

The levels of cytokines in peripheral blood were detected by CBA (cytomic beam array system) flow cytometry according to the manufacturer’s instructions.

### Statistical Analysis

Post hoc power analyses were conducted with GPOWER (Faul, Erdfelder, Lang, & Buchner, 2007) in order to estimate the probability of occurrence of effects in the sample. The optimal cut-off values of interleukin 10, interleukin 6, and interferon gamma levels were determined by receiver operating characteristic (ROC) curve analysis. They were associated with survival status of patients. Through the normal distribution test, the continuous variables included in this study all conform to the normal distribution. Continuous variables were grouped according to the usual clinical threshold and were presented as frequencies and percentages (n, %) in company with categorical variables. All hierarchical and categorical variables were compared by Pearson’s chi-square test. Among them, histological subtypes were performed bonferroni-post-hoc-correction. The survival curve was attained *via* Kaplan-Meier method and the log-rank test. Univariate and multivariate logistic regression models were used to evaluate the relevance between clinical variables and complete remission (CR) and 1-year recurrence or progression. Cox proportional hazards regression model was used to analyze univariate and multivariate of OS. All statistical analyses were calculated by statistical software package SPSS 23.0. In all comparisons, p<0.05 was considered to be statistically significant.

## Results

### Patients Characteristics and Clinical Outcomes According to the Different Levels of IL-10

According to the criteria, we selected 205 patients with PTCL from January 2014 to June 2019. **Table 1** lists the clinical characteristics and laboratory data of all enrolled patients. The median age at diagnosis was 56 years (range: 18–79 years), and the ratio of male to female was 1.8:1. Eighty point five percent of these patients were in stage III-IV, and 55.1% of them had B symptoms. ENKTL accounted for 33.6% of all pathological subtypes, PTCL-NOS, AITL and ALCL accounted for 21.8, 27.5, and 12.7% respectively. In addition, there were 68 cases of bone marrow involvement, and 24 cases of HPS. More than one extranodal location was involved in 98 patients, and 120 patients were infected with EBV at the first diagnosis. The serum LDH levels were increased in 66.3% of the patients, meanwhile the levels of β2-MG were increased in 63.9%. According to the IPI risk classification, 54.1% of patients (n = 111) were at high or high-moderate risk. **Table 1** shows the clinical characteristics of patients with different IL-10 levels. It shows that patients with IL-10 levels exceeding 3.6 pg/ml have higher IPI scores and ECOG scores, and the proportion of B symptoms and HPS were significantly higher than those with IL-10 levels less than 3.6 pg/ml. Patients with IL-10 levels higher than 3.6 pg/ml had higher proportion of LDH, β2-MG, and SF. However, the different subtypes of PTCL did not show statistical significance in the stratification of IL-10 in our study, either by Pearson’s chi square test (P = 0.287) or performing bonferroni-post-hoc-correction(P = 0.288).

**Table 1:** Analysis of clinical and laboratory characteristics of IL-10 stratification in 205 patients with PTCL.

characteristic	total	IL-10<3.6pg/ml	IL-10≥3.6pg/ml	
	(n=205,%)	(n=108, %)	(n=97, %)	p Value
Age				0.120
<60years	127(62.0)	72(66.7)	56(57.7)	
≥60years	78(38.0)	36(33.3)	41(42.3)	
Sex				0.009^*^
Male	132(64.4)	61(56.5)	71(73.2	
Female	73(35.6)	47(43.5)	26(26.8)	
IPI				0.000^*^
0-2	94(45.9)	70(64.8)	24(24.7)	
3-5	111(54.1)	38(35.2)	73(75.3)	
ECOG				0.000^*^
0-2	154(75.1)	94(87.0)	60(61.9)	
3-5	51(24.9)	14(13.0)	37(38.1)	
Stage				0.000*
I-II	40(19.5)	36(33.3)	4(4.1)	
III-IV	165(80.5)	72(66.7)	93(95.9)	
B symptoms				0.000*
Yes	113(55.1)	45(41.7)	68(70.1)	
No	92(44.9)	63(58.3)	29(29.9)	
Histological				0.287
subtype				
PTCL,NOS	57(27.8)	36(33.3)	23(23.7)	
ENKTL	69(33.6)	36(33.3)	31(32.0)	
AITL	44(21.5)	20(18.5)	24(24.7)	
ALCL,ALK+	16(7.8)	8(7.4)	8(8.3)	
ALCL,ALK-	10(4.9)	6(5.6)	4(4.1)	
Others^#^	9(4.4)	2(1.9)	7(7.2)	
Bone marrow Involvement				0.000*
Yes	68(33.2)	23(78.7)	45(46.4)	
No	137(66.8)	85(21.3)	52(53.6)	
Albumin(g/l)				0.000*
<35	58(28.3)	12(11.1)	46(47.4)	
≥35	147(71.7)	96(88.9)	51(52.6)	
HPS				0.000*
Yes	24(11.7)	3(2.8)	21(21.6)	
No	181(88.3)	105(97.2)	76(78.4)	
Elevated IL-6 level				0.000*
Yes	139(67.8)	60(55.6)	79(81.4)	
No	66(32.2)	48(44.4)	18(18.6)	
Elevated IFN γ level				0.000*
Yes	78(38.0)	23(21.3)	55(56.7)	
No	127(62.0)	85(78.7)	42(43.3)	
EBV				0.028*
Negative	85(41.5)	52(48.1)	33(34.0)	
Positive	120(58.5)	56(51.9)	64(66.0)	
Extra-nodal Involvement				0.000*
≤1	107(52.2)	71(65.7)	36(37.1)	
>1	98(47.8)	37(34.3)	61(62.9)	
Elevated LDH level				0.000*
Yes	136(66.3)	60(55.6)	76(78.4)	
No	69(33.7)	48(44.4)	21(21.6)	
Elevated β2-MG level				0.000*
Yes	131(63.9)	55(50.9)	76(78.4)	
No	74(36.1)	53(49.1)	21(21.6)	
Elevated SF level				0.004*
Yes	108(52.7)	47(43.5)	61(62.9)	
No	97(47.3)	61(56.5)	36(37.1)	
Attainment of CR				0.000*
Yes	70(34.1)	56(51.9)	14(14.4)	
No	135(65.9)	52(48.1)	83(85.6)	
Recurrence/progress in one year				0.000*
Yes	67(32.7)	19(17.6)	48(49.5)	
No	138(67.3)	89(82.4)	49(50.5)	

^#^others: 3 cases of MEITL, 1 case of T-LGLL, 2 cases of SPTCL, 2 cases of MF/SS, 1 case of HSTCL; CR, complete response.

^*^Significantly different.

The classification variables are expressed by frequency and percentage. (n, %).

### Association of Serum Cytokines With Survival Outcome and Early Recurrence

**Figure 1:**
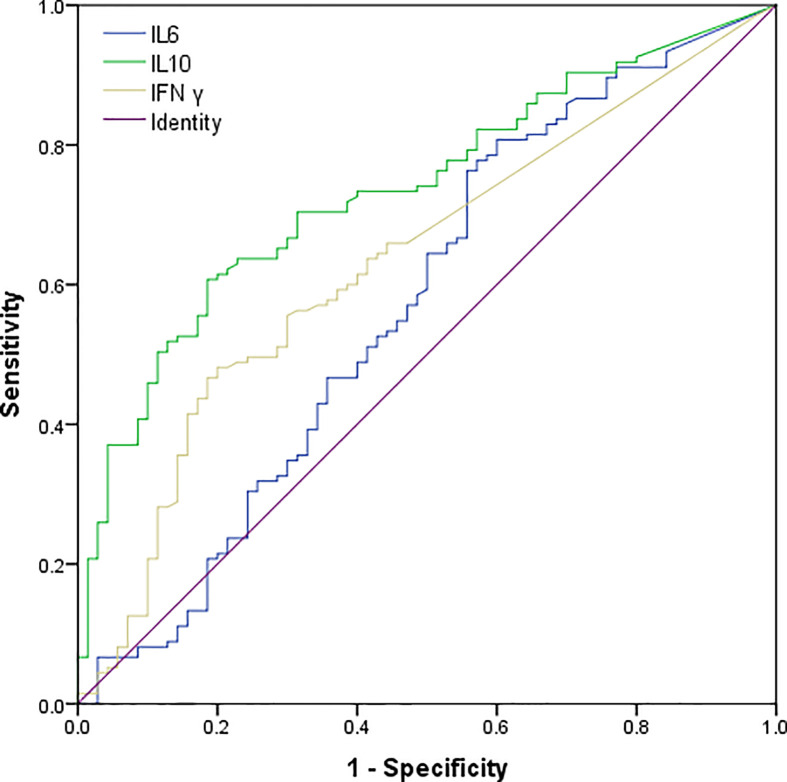
Receiver operating characteristic (ROC) curves of serum IL-10, IL-6, and IFN γ levels of patients with PTCL. The AUC of IL-10, IL-6 and IFN γ were 0.736 (95% CI: 0.668–0.805), 0.572(95% CI: 0.485–0.659), 0.634(95% CI: 0.554–0.713)..

**Figure 2:**
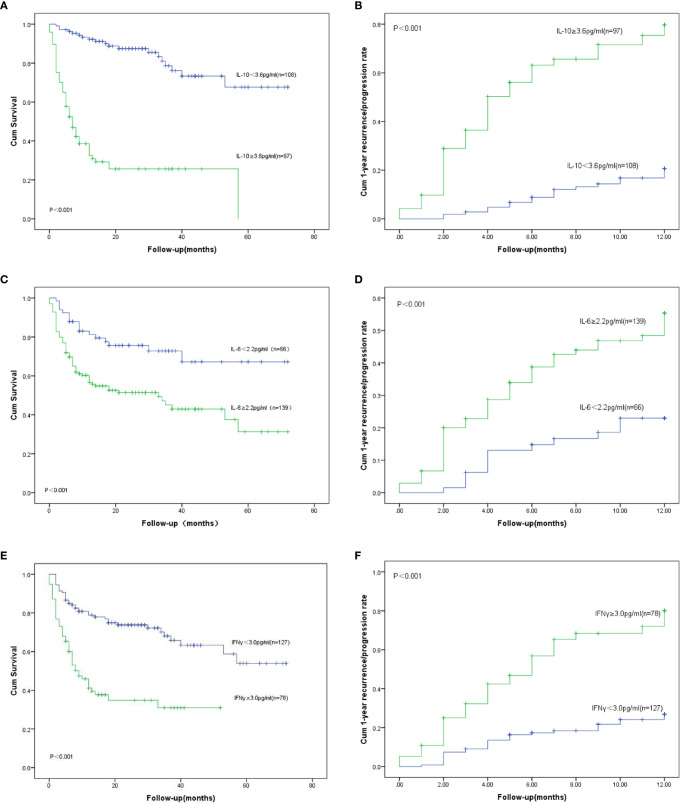
OS: Overall survival. **(A)** OS for different serum levels of IL-10; **(B)** Recurrence/progression in one year for different serum levels of IL-10. **(C)** OS for different serum levels of IL-6; **(D)** Recurrence/progression in one year for different serum levels of IL-6; **(E)** OS for different serum levels of IFN γ; **(F)** Recurrence/progression in one year for different serum levels of IFN γ.

We had analyzed six cytokines: IL-4, IL-6, IL-10, TNFα, IFNγ, and IL-17A. Only IL-6, IL-10, and IFNγ were found to have statistical significance on the survival and prognosis of PTCL patients and the median value and range of serum levels of these three cytokines were as follows: IL-10 (3.13 pg/ml, range: 0.1–8536.4 pg/ml), IL-6 (6.65 pg/ml, range: 0.1–815.2 pg/ml), and IFN-γ (1.44 pg/ml, range: 0–1330.9 pg/ml), respectively. According to the ROC curve, the cutoff values of IL-6, IL-10, and IFNγ were determined to be 2.2 pg/ml, 3.6 pg/ml and 3.0 pg/ml, respectively. The area under the curve are 0.572, 0.736, 0.634 for the IL-6, IL-10 and IFNγ (**Figure 1**). Therefore, the high group and low group were defined as being greater than or equal to the cutoff value and less than the cutoff value, respectively. Post hoc analysis demonstrated sufficient power to distinguish the significant differences (power = 0.945). Patients with IL-10 ≥3.6 pg/ml, only 14.4% of them achieved CR after treatment, but the rate of recurrence or progression within one year was nearly half, reaching 49.5%. In the group of patients with IL-10 <3.6 pg/ml, more than half of them achieved CR (51.9%) after treatment, and only 17.6% of patients relapsed or progressed within one year. Therefore, it is not difficult to speculate that patients with IL-10 ≥3.6 pg/ml may have poor therapeutic effect and are prone to early recurrence (**Table 1**). Also, the patients with IL-10 ≥3.6 pg/ml achieved lower OS and higher early recurrence or progression rate (**Figures 2A, B**, P value < 0.001). In addition, the cumulative survival rate and cumulative 1-year recurrence or progression rate between the high IL-6 and low IL-6 groups and the high IFNγ and low IFNγ groups were statistically significant (**Figures 2C–F**, all P value<0.001). To our disappointment, they were not independent factors (**Tables 3**, **4**).

In univariate logistic regression analysis, lower CR rate was related to male, first diagnosis older than 60 years, stage III-IV, B symptoms, bone marrow involvement, IPI ≥3, ECOG ≥3, EBV infection, Extra-nodal ≥1, IL-10 ≥3.6 pg/ml, IL-6 ≥2.2 pg/ml, IFN γ ≥3.0 pg/ml, decreased LDH and decreased β2-MG (**Table 2**). Nevertheless, in the multivariate logistic regression analysis, only ECOG ≥3, EBV infection, IL-10 ≥3.6 pg/ml were statistically significant (**Table 2**).

**Table 2:** Univariate and multivariate logistic regression models of complete response (CR) in PTCL patients.

Parameters	Univariate analysis			Multivariate analysis		
	OR	95% CI	p Value	OR	95%CI	p Value
Sex, Male	0.472	0.260-0.858	0.014*			
Age, ≥60years	2.273	1.204-4.290	0.011*			
Stage,III-IV	5.107	2.447-10.658	<0.001*			
B symptoms	2.128	1.184-3.827	0.012*			
Bone marrow Involvement	3.014	1.507-6.030	0.002*			
IPI,3-5	3.474	1.894-6.371	<0.001*			
ECOG,3-5	8.812	3.024-25.680	<0.001*	4.359	1.317-14.428	0.016*
EBV,Positive	2.667	1.473-4.827	0.001*	2.419	1.209-4.840	0.013*
Extra-nodal, >1	2.119	1.169-3.841	0.013*			
IL-10≥3.6pg/ml	6.385	3.233-12.609	<0.001*	2.973	1.344-6.578	0.015*
IL-6≥2.2pg/ml	2.271	1.236-4.175	0.008*			
IFN-γ≥3.0pg/ml	3.606	1.834-7.089	<0.001*			
Elevated LDH	2.230	1.220-4.078	0.009*			
Elevated β2-MG	2.045	1.127-3.170	0.019*			

OR, odds ratio; CI, confidence interval.

^*^Significantly different.

In univariate logistic regression analysis, higher 1-year recurrence or progression rate was associated with stage III-IV, B symptoms, bone marrow involvement, IPI ≥3, ECOG ≥3, decreased albumin, Extra-nodal ≥1, IL-10 ≥3.6 pg/ml, IL-6 ≥2.2 pg/ml, IFN γ ≥3.0 pg/ml, decreased SF (**Table 3**, **Figures 2B, D, E**). However, only bone marrow involvement (OR = 3.799, 95% CI 1.630–8.854, p = 0.002), ECOG ≥3(OR = 5.873, 95% CI 2.464–13.997, p < 0.001), IL-10 ≥3.6 pg/ml(OR = 2.008, 95% CI 0.876–4.601, p = 0.009) had statistically significant in the multivariate logistic regression analysis (**Table 3**).

**Table 3:** Univariate and multivariate logistic regression models for recurrence or progression within one year in PTCL patients.

Parameters	Univariate analysis			Multivariate analysis		
	OR	95%CI	p Value	OR	95%CI	p Value
Stage,III-IV	5.559	1.888-16.364	0.002*			
B symptoms	2.106	1.145-3.873	0.017*			
Bone marrow Involvement	5.235	2.774-9.880	<0.001*	3.799	1.630-8.854	0.002*
IPI,3-5	3.008	1.605-5.636	0.001*			
ECOG,3-5	8.340	4.108-16.931	<0.001*	5.873	2.464-13.997	<0.001*
Decreased albumin	0.228	0.119-0.435	<0.001*			
Extra-nodal, >1	2.460	1.349-4.485	0.003*			
HPS	4.135	1.703-10.037	0.002*			
IL-10≥3.6pg/ml	4.589	2.431-8.663	<0.001*	2.008	0.876-4.601	0.009*
IL-6≥2.2pg/ml	2.590	1.291-5.194	0.007*			
IFN γ≥3.0pg/ml	3.536	1.920-6.512	<0.001*			
Elevated SF	1.833	1.009-3.330	0.047*			

OR, odds ratio; CI, confidence interval.

^*^Significantly different.

### Univariate and Multivariate Cox Proportional Hazards Regression Analysis of OS in PTCL Patients

In our study, recurrence or progression occurred in 67 patients within one year, accounting for 32.7%. The median survival time in the groups with IL-10 ≥3.6 pg/ml and IL-10 <3.6 pg/ml were 6 months and 26 months (**Figure 2A**), respectively. Compared with patients with IL-10 <3.6 pg/ml, 1-year OS and 2-year OS were lower in patients with IL-10 ≥3.6 pg/ml (80.6 vs. 20.6%, p < 0.001; 54.6 vs. 11.3%, p < 0.001, **Figure 2A**). The univariate analysis showed that stage III-IV, B symptoms, bone marrow involvement, IPI ≥3, ECOG ≥3, decreased albumin, EBV infection, Extra-nodal ≥1, HPS, IL-10 ≥3.6 pg/ml, IL-6 ≥2.2 pg/ml, IFN γ ≥3.0 pg/ml, decreased LDH and decreased serum ferritin were prognostic indicators of OS (**Table 4**, **Figures 2C, E**). Then, multivariate analysis was showed that patients with poor OS had ECOG ≥3 (HR = 4.314, 95% CI 2.462–7.559, p < 0.001), Extra-nodal ≥1 (HR = 2.126, 95% CI 1.142–3.958, p = 0.017), IL-10 ≥3.6 pg/ml (HR = 6.428, 95% CI 3.369–12.266, p < 0.001), and IL-6 ≥2.2 pg/ml (HR = 2.050, 95% CI 1.122–3.745, p = 0.020).

**Table 4:** Univariate and multivariate Cox proportional hazard regression models for overall survival (OS) in PTCL patients.

Parameters	Univariate analysis			Multivariate analysis		
	HR	95%CI	p Value	HR	95%CI	p Value
Stage,III-IV	3.079	1.486-6.382	0.002*			
B symptoms	2.483	1.547-3.985	<0.001*			
Bone marrow Involvement	2.851	1.860-4.369	<0.001*			
IPI,3-5	2.806	1.749-4.499	<0.001*			
ECOG,3-5	4.522	2.933-6.971	<0.001*	4.314	2.462-7.559	<0.001*
Decreased albumin	0.344	0.224-0.527	<0.001*			
EBV,Positive	1.671	1.060-2.637	0.027*			
Extra-nodal, >2	3.004	1.903-4.742	<0.001*	2.126	1.142-3.958	0.017*
HPS	3.678	2.176-6.218	<0.001*			
IL-10≥3.6pg/ml	8.034	4.741-13.613	<0.001*	6.428	3.369-12.266	<0.001*
IL-6≥2.2pg/ml	2.516	1.477-4.283	0.001*	2.050	1.122-3.745	0.020*
IFN-γ≥3.0pg/ml	3.249	2.095-5.037	<0.001*			
Elevated LDH	2.032	1.220-3.387	0.006*			
Elevated SF	1.605	1.041-2.476	0.032*			

HR, hazard ratio; CI, confidence interval.

^*^Significantly different.

Thus, it can be seen that among the three cytokines included in our studies, only IL-10 was significant and independent prognostic factor in both univariate and multivariate analyses of treatment response, survival, and early recurrence in PTCL patients. IL-6 had an independent effect on OS in multivariate analyses as well. The results of Cox regression analysis were present in **Table 4**.

## Discussion

Benefit from the oceans of studies about tumor micro-environment, tremendous progress has been made in predicting the prognosis of PTCL. Nevertheless, poor response, high mortality, and relapse remain primary challenges, so new prognostic factors need to be explored to further predict and improve the prognosis of PTCL patients. According to the established tumor models, Sato T et al. found that IL-10 has tumor-promoting and anti-tumor effects *in vivo* (14). Herein, we retrospectively analyzed six cytokines of 205 patients with PTCL and discovered that patients with IL-10 ≥3.6 pg/ml had both low CR rate, low OS rate but high 1-year recurrence or progression rate as an independent effect in multivariate analyses. Therefore, from our retrospective clinical studies, IL-10 may play a role as pro-tumor effects *in vivo*.

C. Andrew Stewart et al. observed that the majority of tumor- associated IL-10 was produced by activated Tregs. Components of the type I IFN signaling pathway, including Ifnar1, Stat1 and Stat2, were essential for the accumulation and activation of Tregs and production of IL-10 (15). In cancer-related preclinical and therapeutic models, Tregs limits the production of Th1 responses that drive CD8 + T cells and IFN γ dependent anti-tumor immunity (16, 17). Th17 cells occur in most human tumors frequently. Th17 related inflammation may lead to tumor growth or autoimmunity. The production of IL-10 limits the quantities and activity of Th17 cells in tumors, which promotes the growth of tumors (18).

The poor response of immunotherapy in most tumors were bound up with tumor-associated macrophages (TAM), Tregs and myeloid-derived suppressor cells (MDSCs). MDSCs can induce the production of Tregs and lead Tregs to tumor tissues, thus promoting the production of IL-10 (19). TAM were related to the poor clinical prognosis of various human tumors by promoting angiogenesis, local invasion and metastasis (20–23). It has been confirmed that the growth and survival of malignant T cells depend on alternately activated (M2) macrophages in the micro-environment, and the growth of alternately activated (M2) macrophages was affected by the presence of TAMs (4, 24). Sam T. Hwang found that alternately activated (M2) macrophages can affect the polarization of undifferentiated macrophages in the way specified (25). In a mouse model study, M2 macrophages can increase the secretion of IL-10 by macrophages which is necessary for the maximum growth of human cutaneous T-cell lymphoma (25). Kim SJ and Ham JS analyzed the serum cytokines and CD68- and CD163- positive macrophage in tumor tissue of 37 AITL patients after CHOP chemotherapy, and found that high IL-10 and M2 macrophage tissue infiltration all indicated low OS and poor response to treatment (26). In subsequent studies, this team further tested 34 cytokines in 121 patients with PTCL, and analyzed the correlation between overall survival rate and complete remission rate. They found that patients with AITL had higher serum cytokine levels, and IL-10 levels higher than 3.8 pg/ml were associated with adverse outcomes, which was very close to our cut-off value. In addition to IL-10, IFN γ, IL-8, IL-17, IL-23, monocyte chemoattractant protein-1 and macrophage inflammatory protein-1β, and RANTES negatively also have negative effects on clinical prognosis in patients with ALK- anaplastic large cell lymphoma (27). Li Li and Zhang Jun found that the released IL-10 made the JAK2/STAT3 pathway sensitive, leading to STAT3-induced PD-L1 expression (28). At the same time, they confirmed PD-L1 signaling network in 2 main 428 and 350 DLBCL cohorts, and showed a significant correlation among IL-10, STAT3, and PD-L1 (28). H. Dean et al. conducted genetic analysis of 20 single nucleotide polymorphisms (SNPs) in IL-10 and TNF/LTA loci in three independent case-control studies (2,635 cases and 4,234 controls), discovered IL-10 rs1800896 was related to DLBCL, as well as T-cell lymphoma (29). It was confirmed that genetic variations of immune-related genes (such as IL-10) were related to the occurrence of T-cell lymphoma in Asian populations (29). To sum up, it had been proved that IL-10 was related to the prognosis of malignant T-cell lymphoma in both gene and molecular studies and animal experimental studies. It promoted tumor cells to evade immune surveillance by regulating antigen presentation and immune cell differentiation (30). Mona R. Hassuneh et al. used LSA (a T-cell lymphoma cell line expressing IL-10) to study the role of IL-10 in tumorigenesis. Interestingly, the administration of anti-IL-10 antibodies significantly inhibited the growth of tumors in LSA (31).

Previously, stage was well recognized as having a prognostic impact in PTCL, and it had been included in previously reported indices in subsequent studies (32), indicating that it was highly significant predictor of OS. In our study, stage III–IV was associated with low response rate, early recurrence and poor OS, which was consistent with previous studies. In **Table 1**, it is easy to find that in patients with IL-10 levels higher than 3.6 pg/ml, the proportion of stage III–IV is significantly higher than that of stage I–II (95.9 vs 4.1%). It is further suggested that the increase of IL-10 levels might be related to distant metastasis and immune escape.

IL-10 has strong immunosuppression. It inhibits the proliferation of T cells (33). Thus, overexpression of IL-10 by PTCL lymphoma cells may damage the host’s immune system, resulting in immunosuppression and tumor escape. At the same time, it will also provide a selective survival advantage for the PTCL cells in the host. What is more, serum IL-10 concentration is also an important prognostic factor for various tumors, such as diffuse large B-cell lymphoma (DLBCL) and adult T-cell leukemia/lymphoma (ATL) (12, 20, 34). Our study proved that IL-10 plays an important role in the treatment response, survival and early recurrence or progression of PTCL patients. In multivariate regression analysis, patients with IL-10 ≥3.6 pg/ml had a lower CR rate and a higher recurrence rate. In univariate and multivariate survival analysis, patients with IL-10 ≥3.6 pg/ml had poorer OS. To the best of our knowledge, this retrospective clinical study is the first to confirm the effect of IL-10 on the treatment response, survival, and early recurrence of Chinese PTCL patients. Insaki Atsushi et al. analyzed the serum IL-10 levels in 94 cases of ATL and found that high IL-10 levels were an important adverse prognostic factor in ATL. However, the critical value of IL-10 in our study is different from the results of Atsushi Inagaki et al. (34). They mainly target ATL patients between the ages of 38–89 and the number of cases in their study is less than 100, however our study contained the five major PTCL subtypes and the five rare subtypes of MEITL, T-LGLL, SPTCL, MF/SS, and HSTCL with age range of 18–79 years old, and we have more than twice as many cases as they have. Perhaps, the different number of cases, disease types and inclusive criteria that led to the different cutoff values. Similarly, IL-10 is still an independent prognostic factor for survival and early recurrence in patients with PTCL.

In addition, we were surprised to find that there was a gender difference between the high IL-10 group and the low IL-10 group. The proportion of female patients was significantly lower than the male patients in high IL-10 group (26.8 vs 73.2%), and the treatment response of female patients was better than that of the male (**Table 2**). It aroused our interest, and we specially reviewed some similar previous studies. Previous studies had shown the differences in immune function between age and gender. These differences were believed to be caused by hormone discrepancies, especially the loss of estradiol caused the reversal of immune response in elderly females (35). Interestingly, the inhibitory effect of female derived MDSCs on T cells proliferation were stronger than that of male. Transplantation of female derived MDSCs significantly increased the frequency and absolute number of Tregs and CD4 + IL-10 + T cells (36). However, in tumor environment, MDSCs not only have characteristics of M2 macrophages (such as the expression of arginase-1 and NOS2), but also play a role as the progenitor cells of tumor associated macrophages. Moreover, MDSCs can further regulate the cytotoxicity mediated by macrophages (37, 38).

Hemophagocytic syndrome (HPS) is an immune-mediated life-threatening disease that affects 1% of adults with hematological cancers, and its prevalence rises to 20% in patients with NHL, especially PTCL (39). Qi An et al. found that median serum concentrations of IFN γ and IL-10 were significantly higher in children with HPS compared to healthy controls (40). In our study, there were 24 patients with HPS, accounting for 11.7%, and the median serum of IL-10 and IFN γ were higher than the patients without HPS (54.02 vs. 2.70 pg/ml, p < 0.001; 12.42 vs. 1.02pg/ml, p < 0.001). Importantly, although the OS of PTCL patients with HPS decreased significantly, HPS was not an independent predictor for OS of PTCL patients after univariate and multivariate analysis in our study.

Our study still has some limitations. Firstly, the retrospective study may be biased in the selection of patients. Secondly, the dynamic changes of patients during treatment did not take into account during analysis. Thirdly, due to the limitation of the number of cases, the conclusions of this study are only verified in three common subtypes of PTCL-NOS, ENKTL, and AITL. Therefore, more in-depth studies are needed to confirm the role of IL-10 in the prognosis of patients with PTCL.

## Conclusions

All in all, increased serum levels of IL-10 at diagnosis is related to the survival and early recurrence of PTCL patients. IL-10 ≥3.6 pg/ml is significantly correlated with lower CR rate, higher recurrence rate and lower OS rate. Furthermore, univariate and multivariate analysis indicated that IL-10 ≥3.6 pg/ml is an independent prognostic factor. In short, the serum level of IL-10 in the peripheral blood at the initial stage can be added to the prognostic tool to play its prognostic role in PTCL. In addition, the determination of serum cytokine levels is quick and convenient. Only a small amount of serum samples can be used to complete the detection. This is very useful to help us formulate different treatment strategies for individuals with PTCL, because it can predict the prognosis before treatment. In the near future, IL-10 inhibitors or antagonists may become a new method of Immunotargeting therapy for PTCL patients.

## Data Availability Statement

The raw data supporting the conclusions of this article will be made available by the authors, without undue reservation.

## Ethics Statement

Written informed consent was obtained from the individual(s) for the publication of any potentially identifiable images or data included in this article.

## Author Contributions

WX designed the study. YZ, YLZ, LS, YS, HS, MZ, XY, and JJ collected the patients’ material. YZ and YLZ analyzed data and wrote the paper. All authors contributed to the article and approved the submitted version.

## Conflict of Interest

The authors declare that the research was conducted in the absence of any commercial or financial relationships that could be construed as a potential conflict of interest.

## References

[1] American Society of Hematology A clinical evaluation of the Inernational Lymphoma Study Group classification of non-Hodgkin’s lymphoma. The Non-Hodgkin’s Lymphoma Classification Project. Blood (1997) 89:3909–18. 10.1182/blood.V89.11.3909 9166827

[2] Shi Y Current status and progress of lymphoma management in China. Int J Hematol (2018) 107(4):405–12. 10.1007/s12185-018-2404-8 29388166

[3] Swerdlow SH Campo E Harris NL World Health Organization classification of tumors of haematopoietic and lymphold tissues. Lyon, France: IARC (2016).

[4] Wilcox RA Wada DA Ziesmer SC Elsawa SF Comfere NF Dietz AB Monocytes promote tumor cell survival in T-cell lymphoproliferative disorders and are impaired in their ability to differentiate into mature dendritic cells. Blood (2009) 114(14):2936–44. 10.1182/blood-2009-05-220111 PMC2756204 19671921

[5] Staples KJ Smallie T Williams LM Foey A Burke B Foxwell BMJ IL-10 Induces IL-10 in Primary Human Monocyte-Derived Macrophages via the Transcription Factor Stat3. J Immunol (2007) 178:4779–85. 10.4049/jimmunol.178.8.4779 17404258

[6] Iqbal J Wright G Wang C Gene expression signatures delineate biological and prognostic subgroups in peripheral T-cell lymphoma. Blood (2014) 123:2915–23. 10.1182/blood-2013-11-536359 PMC4014836 24632715

[7] Wang T Feldman AL Wada DA Lu Y Polk A Briski R GATA-3 expression identifies a high-risk subset of PTCL, NOS with distinct molecular and clinical features. Blood (2014) 123:3007–15. 10.1182/blood-2013-12-544809 PMC4014843 24497534

[8] Xua G Wang F Associations of polymorphisms in interleukins with susceptibility to breast cancer: Evidence from a meta-analysis. Cytokine (2020) 130:154988. 10.1016/j.cyto.2020.154988 32163880

[9] Lombardi Pereira AP Paiva Trugilo K Okuyama NCM Mota Sena M D'Oliveira Couto-Filho J Watanabe MAE IL-10 c.-592C>A (rs1800872) polymorphism is associated with cervical cancer. J Cancer Res Clin Oncol (2020) 146(8):1971–8. 10.1007/s00432-020-03256-0 PMC11804423 32447484

[10] Zhong-Wu LU Hu J-Q Liu W-L Wen D Wei W-J Wang Y-L IL-10 restores MHC class I expression and interferes immunity in papillary thyroid cancer with Hashimoto’s thyroiditis. Endocrine Soc (2020). 10.1210/endocr/bqaa062 PMC7469947 32348468

[11] Sakai Y Rezano A Okada S Ohtsuki T Kawashima Y Tsukamoto T A Novel Cytological Model of B-Cell/Macrophage Biphenotypic Cell Hodgkin Lymphoma in Ganp-Transgenic Mice. Cancers (Basel) (2020) 12(1):204. 10.3390/cancers12010204 PMC7017268 31947626

[12] Yi JH Yoon SE Ryu KJ Ko YH Kim WS Kim SJ Pre-treatment serum IL-10 predicts the risk of secondary central nervous system involvement in patients with diffffuse large B-cell lymphoma. Cytokine (2020) 129:155048. 10.1016/j.cyto.2020.155048 32135464

[13] Altman DG McShane LM Sauerbrei W Taube SE Reporting Recommendations for Tumor Marker Prognostic Studies (REMARK): Explanation and Elaboration. PloS Med (2012) 9(5):e1001216. 10.1371/journal.pmed.1001216 22675273 PMC3362085

[14] Sato T Terai M Tamura Y Alexeev V Mastrangelo MJ Selvan SR Interleukin 10 in the tumor microenvironment: a target for anticancer immunotherapy. Immunol Res (2011) 51:170–82. 10.1007/s12026-011-8262-6 22139852

[15] Andrew Stewart C Muller W Trinchieri G Interferon-dependent IL-10 production by Tregs limits tumor Th17 inflammation. J Clin Invest (2013) 123(11):4859–74. 10.1172/JCI65180 PMC3809773 24216477

[16] Teng MW Ngiow SF von Scheidt B McLaughlin N Sparwasser T Smyth MJ Conditional regulatory T-cell depletion releases adaptive immunity preventing carcinogenesis and suppressing established tumor growth. Cancer Res (2010) 70(20):7800–9. 10.1158/0008-5472.CAN-10-1681 20924111

[17] Teng MW Ritchie DS Neeson P Smyth MJ Biology and clinical observations of regulatory T cells in cancer immunology. Curr Top Microbiol Immunol (2011) 344:61–95. 10.1007/82_2010_50 20512555

[18] Wilke CM Wang L Wei S Kryczek I Zou W Endogenous interleukin-10 constrains Th17 cells in patients with inflammatory bowel disease. J Transl Med (2011) 9:217. 10.1186/1479-5876-9-217 22176654 PMC3264534

[19] Du J Sun X Song Y The study of CD14+HLA-DR-/low myeloid-drived suppressor cell (MDSC) in peripheral blood of peripheral T-cell lymphoma patients and its biological function. Cell Mol Biol (Noisy-le-grand) (2017) 63(3):62–7. 10.14715/cmb/2017.63.3.12 28466815

[20] Malesci A Bianchi P Celesti G Basso G Marchesi F Grizzi F Tumor-associated macrophages and response to 5-fluorouracil adjuvant therapy in stage III colorectal cancer. Oncoimmunology (2017) 6(12):e1342918. 10.1080/2162402X.2017.1342918 29209561 PMC5706531

[21] Qiu SQ Waaijer SJH Zwager MC de Vries EGE van der Vegt B Schröder CP Tumor-associated macrophages in breast cancer: Innocent bystander or important player? Cancer Treat Rev (2018) 70):178–89. 10.1016/j.ctrv.2018.08.010 30227299

[22] Futagbi G Gyan B Nunoo H Tetteh JKA Welbeck JE Renner LA High Levels of IL-10 and CD4+CD25hi+ Treg Cells in Endemic Burkitt’s Lymphoma Patients. Biomedicines (2015) 3:224–36. 10.3390/biomedicines3030224 PMC5344238 28536409

[23] Ryder M Ghossein RA Ricarte-Filho JC Knauf JA Fagin JA Increased density of tumor-associated macrophages is associated with decreased survival in advanced thyroid cancer. Endocr Relat Cancer (2008) 15(4):1069–74. 10.1677/ERC-08-0036 PMC2648614 18719091

[24] Wu X Schulte BC Zhou Y Depletion of M2-like tumor-associated macrophages delays cutaneous T-cell lymphoma development in vivo. J Invest Dermatol (2014) 134:2814–22. 10.1038/jid.2014.206 24780929

[25] Wu X Hsu DK Wang K-H Huang Y Mendoza L Zhou Y IL-10 is overexpressed in human cutaneous T-cell lymphoma and is required for maximal tumor growth in a mouse model. Leuk Lymphoma. (2019) 60(5):1244–52. 10.1080/10428194.2018.1516037 30277131

[26] Ham JS Park HY Ryu KJ Ko YH Kim WS Kim SJ Elevated serum interleukin-10 level and M2 macrophage infiltration are associated with poor survival in angioimmunoblastic T-cell lymphoma. Oncotarget (2017) 8(44):76231–40. 10.18632/oncotarget.19301 PMC5652701 29100307

[27] Yi JH Ryu KJ Ko YH Kim WS Kim SJ Profiles of serum cytokines and their clinical implications in patients with peripheral T-cell lymphoma. Cytokine (2019) 113:371–9. 10.1016/j.cyto.2018.10.009 30327172

[28] Li L Zhang J Pham LV B-cell receptor-mediated NFATc1 activation induces IL-10/STAT2/PD-L1 signaling in diffuse large B-cell lymphoma. Blood (2018) 132(17):1805–17. 10.1182/blood-2018-03-841015 PMC6634963 30209121

[29] Dean Hosgood III H Au W-Y Kim HN Liu J Hu W Tse J IL-10 and TNF variants and risk of non-Hodgkin lymphoma among three Asian populations. Int J Hematol (2013) 97:793–9. 10.1007/s12185-013-1345-5 PMC4241501 23640160

[30] Mantovani A Sozzani S Locati M Macrophage polarization: tumor-associated macrophages as a paradigm for polarized M2 mononuclear phagocytes. Trends Immunol (2002) 23:549–55. 10.1016/S1471-4906(02)02302-5 12401408

[31] Hassuneh MR Nagarkatti M Nagarkatti PS Role of interleukin-10 in the regulation of tumorigenicity of a T cell lymphoma. Leuk Lymphoma (2013) 54(4):827–34. 10.3109/10428194.2012.726721 PMC4156858 22946665

[32] Weisenburger DD Savage KJ Harris NL Gascoyne RD Jaffe ES MacLennan KA Peripheral T-cell lymphoma, not otherwise specifified: a report of 340 cases from the International Peripheral T-cell Lymphoma Project. Blood (2011) 117:3402–8. 10.1182/blood-2010-09-310342 21270441

[33] Taga K Mostowski H Tosato G Human interleukin-10 can directly inhibit T-cell growth. Blood (1993) 81:2964–71. 10.1182/blood.V81.11.2964.bloodjournal81112964 8499633

[34] Inagaki A Ishida T Ishii T Komatsu H Iida S Ding J Clinical signifificance of serum Th1-, Th2- and regulatory T cells-associated cytokines in adult T-cell leukemia/lymphoma: High Interleukin-5 and -10 levels are signifificant unfavorable prognostic factors. Int J Cancer (2006) 118:3054–61. 10.1002/ijc.21688 16425276

[35] Kahlke V Angele MK Ayala A Schwacha MG Cioffi WG Bland KI Immune dysfunction following traumahemorrhage: influence of gender and age. Cytokine (2000) 12:69–77. 10.1006/cyto.1999.0511 10623445

[36] Su N Yue Y Xiong S Monocytic myeloid-derived suppressor cells from females, but not males, alleviate CVB3- induced myocarditis by increasing regulatory and CD4+IL-10+ T cells. Sci Rep (2016) 6:22658. 10.1038/srep22658 26939768 PMC4778123

[37] Youn JI Gabrilovich DI The biology of myeloid-derived suppressor cells: the blessing and the curse of morphological and functional heterogeneity. Eur J Immunol (2010) 40:2969–75. 10.1002/eji.201040895 PMC3277452 21061430

[38] Maeda A Eguchi H Nakahata K Lo P-C Yamanaka K Kawamura T Monocytic MDSCs regulate macrophage-mediated xenogenic cytotoxicity. Transpl Immunol (2015) 33:140–5. 10.1016/j.trim.2015.07.002 26209355

[39] Ramos-Casals M Brito-Zerón P López-Guillermo A Khamashta MA Bosch X Adult haemophagocytic syndrome. Lancet (2014) 383:1503–16. 10.1016/S0140-6736(13)61048-X 24290661

[40] An Q Hu S-Y Xuan C-M Jin M-W Ji Q Wang Y Interferon gamma and interleukin 10 polymorphisms in Chinese children with hemophagocytic lymphohistiocytosis. Pediatr Blood Cancer (2017) 64:9. 10.1002/pbc.26505 28332776

